# Synchronous gangrenous cholecystitis and appendicitis secondary to appendiceal diverticulum perforation

**DOI:** 10.1093/jscr/rjae785

**Published:** 2024-12-12

**Authors:** Sarah Fennelly, Marilla Dickfos, Jyothirmayi Velaga, Nezor Houli

**Affiliations:** Northern Health Acute General Surgery Unit, 185 Cooper Street, Epping 3076, Australia; Northern Health Acute General Surgery Unit, 185 Cooper Street, Epping 3076, Australia; Northern Health Acute General Surgery Unit, 185 Cooper Street, Epping 3076, Australia; Northern Health Acute General Surgery Unit, 185 Cooper Street, Epping 3076, Australia

**Keywords:** general surgery, acute appendicitis, acute cholecystitis, type 2 diabetes, necrotic gallbladder, perforated appendicitis

## Abstract

Acute appendicitis and acute cholecystitis are among the commonest pathologies in acute general surgery. They are characterized by distinct symptoms, clinical examination findings and typical elements of the history which direct further investigations. In the absence of these classic findings, these diagnoses can be missed, particularly where they occur synchronously. Here we present the interesting case of a 63-year-old male who presented to the emergency department with epigastric pain and vomiting with no classical appendicitis or cholecystitis findings who was found to have both appendicitis with a 3 cm collection and gangrenous cholecystitis, managed with urgent laparoscopic appendicectomy and cholecystectomy. This patient had a background of Type 2 Diabetes which may have reduced awareness of symptoms. Given the increasing prevalence of Type 2 Diabetes and the risk of rapid deterioration in these patients, this case demonstrates the importance of prompt assessment of the entire abdomen when these patients present with acute abdomen.

## Introduction

Synchronous appendicitis and cholecystitis are a rare occurrence, with only 21 cases published in the literature to date [1–15]. The oldest of these case reports was published in 1977 [3] and both appendicitis and cholecystitis were diagnosed on visual inspection during laparotomy. With advances in pre-operative diagnostic imaging, more recent cases have been diagnosed with ultrasonography (US) and computed tomography (CT), allowing for laparoscopic surgery, which is the gold standard of treatment for both conditions [16, 17]. Type 2 Diabetes is associated with increased risk of perforated appendicitis [18] and is a risk factor for asymptomatic gangrenous cholecystitis [19], likely due to reduced or atypical symptoms secondary to neuropathy. It is therefore essential not to exclude other potential diagnoses following the diagnoses of either of these two conditions when these patients present with sepsis.

## Case report

A 63-year-old male presented to the emergency department with a 28-hour history of lower central chest/epigastric pain associated with two episodes of vomiting and hyperglycaemia with a blood glucose level of 25 millimoles (normally 7–8 on oral hypoglycaemics). He was tachycardic with a heart rate of 130 bpm on arrival but afebrile. He had a background of Type 2 Diabetes and hypertension but no surgical history. He had a white cell count of 28.1×10^9^/L and a C-reactive protein (CRP) of 119 mg/dl. His bilirubin was slightly raised at 25 mg/dl and alkaline phosophatase (ALP) 179 U/L but his liver function tests were otherwise normal. On examination his abdomen was soft and mildly tender in the epigastrium, which resolved on repeat examination an hour later.

**Figure 1:**
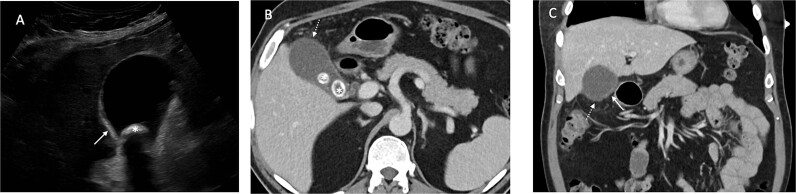
Ultrasound demonstrates gallbladder wall thickening (solid white arrow in A) and gallstone (* in A). Contrast enhanced CT of the abdomen in portal venous phase axial (B) and coronal (C) planes demonstrate gallbladder wall thickening (solid white arrow in C) and area of nonenhancing wall (dashed white arrow in B and C) and gallstones (* in B).

Urgent ultrasound of the upper abdomen and CT abdomen and pelvis were requested. US revealed multiple gallbladder calculi and a 15 mm non-mobile calculus in the neck, and asymmetric thickening of the gallbladder wall suggestive of chronic calculous cholecystitis (Fig. 1). CT showed

**Figure 2:**
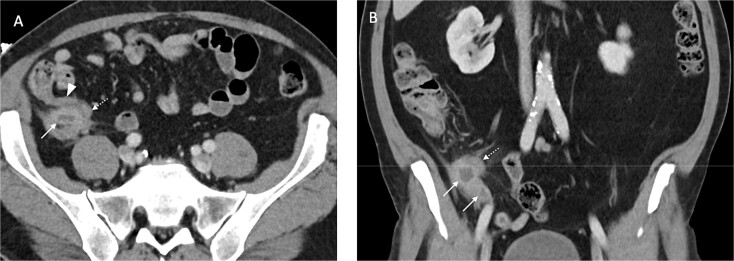
Contrast enhanced axial (A) and coronal (B) CT images in portal venous phase demonstrate periappendiceal abscesses (solid arrows) closely associated with thick-walled and inflamed tip of the appendix (dashed arrows). Note the normal appearing base of the appendix (arrowhead in A).

1. An inflamed appendix with peri-appendiceal stranding and a complex 23 × 20 × 27 mm collection at the apex of the appendix with opposed loop of small bowel with mural thickening
2. Acute cholecystitis with some areas of the gallbladder wall showing no enhancement (Fig. 2)

Insulin-dextrose infusion was commenced by the endocrinology team that evening following labile sugars despite high doses of Novorapid with Optisulin and he was commenced on intravenous piperacillin-tazobactam.

**Figure 3:**
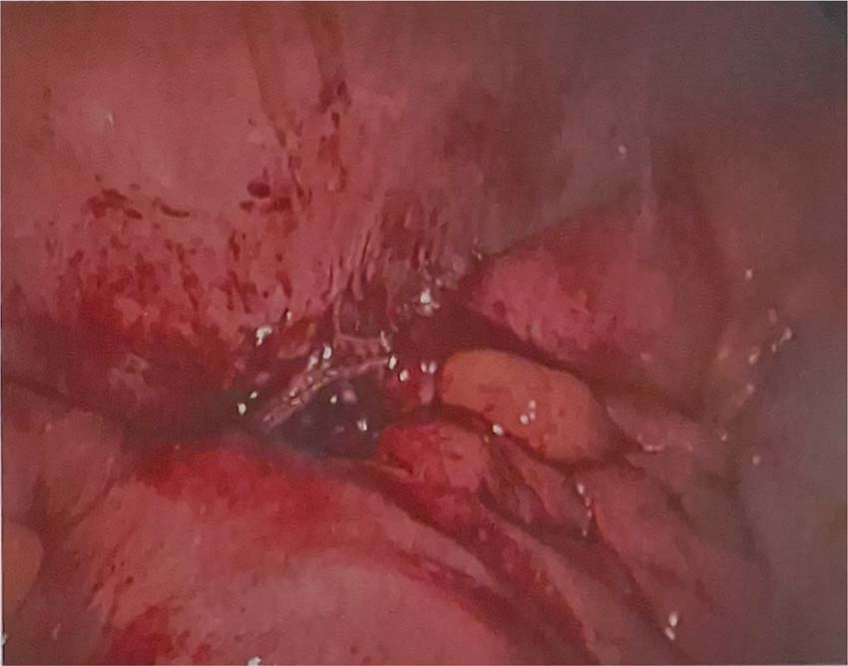
Intraoperative photographs of gangrenous gallbladder.

**Figure 4:**
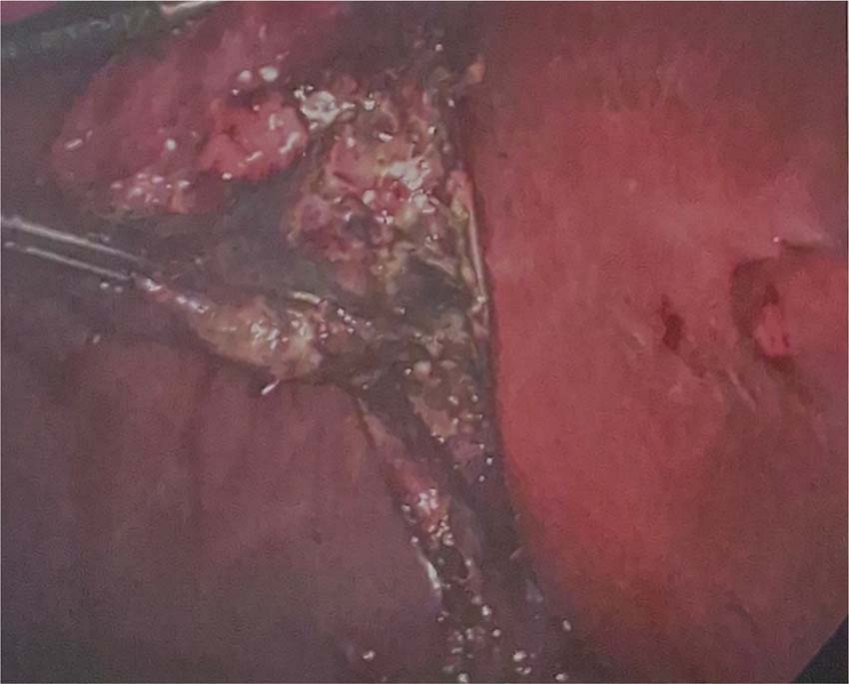
Intraoperative photograph of inflamed, perforated appendix.

He was brought to theatre for a combined laparoscopic appendicectomy, drainage of peri-appendiceal abscess and cholecystectomy with intraoperative cholangiogram. Intraoperative findings included a 2 cm peri-appendiceal abscess cavity (Fig. 3) in keeping with pre-operative imaging. However, the gallbladder was gangrenous with a possible microperforation sealed by omentum (Fig. 4). Based on the intraoperative appearance, it was difficult to ascertain whether the abscess was due to primary appendicitis or whether the abscess was seeded from the cholecystitis and the appendix had become involved collaterally.

The cholecystectomy was performed first due to the evidence of gangrene. The gallbladder was decompressed, draining purulent bile, before dissection of the cystic duct and cystic artery to obtain the critical view of safety. Intraoperative cholangiogram was performed revealing a non-dilated biliary tree, clearly opacified upper ducts, no filling defects, and normal distal tapering with flow into duodenum. The gallbladder was dissected off the liver revealing a completely necrotic posterior wall.

The appendicectomy was then performed, locating the healthy base of the appendix and following its course to the thickened tip. The abscess cavity was opened and thoroughly washed out. The appendix was skeletonised, 2 endoloops were applied and the base was ligated with scissors between the loops. A 15 French Blakes drain was placed through the epigastric port, in the subhepatic space and down the right paracolic gutter. The patient was continued on piperacillin-tazobactam for 48 hours post-operatively.

The insulin infusion was ceased the next morning whilst the drain tube remained in situ for 48 hours. He was stepped down to oral antibiotics and discharged home on D3 post-operation.

Final histology showed gangrenous cholecystitis with evidence of perforation and a calculus lodged in the gallbladder neck. Appendix histology showed features in keeping with a perforated diverticula at the tip of the appendix with associated active inflammation and no fecalith, suggesting that the two pathologies occurred independently of each other.

## Discussion

### Lack of symptoms

This patient presented relatively late with a gangrenous gallbladder and perforated appendicitis. Although his presenting complaints were pain and vomiting, he had no focal tenderness on repeat examination, and was negative for Muphy’s sign, McBurney’s sign, and Rovsing’s sign. On review of the literature, most other cases described symptoms of both appendicitis and cholecystitis, with tenderness over most of the right side. One case described an incidental finding of appendicitis where the patient had denied right iliac fossa (RIF) tenderness [2], although they presented with symptoms consistent with cholecystitis.

Gangrenous cholecystitis and perforation have been reported in an asymptomatic diabetic patient secondary to diabetic neuropathy and/or gallbladder ischemia leading to nerve denervation. In that particular case, the patient presented with 10 days of nausea, vomiting, and hyperglycaemia, with no focal abdominal findings on exam [20]. One case has also been reported of asymptomatic gangrenous cholecystitis in a non-diabetic patient [19]. However, this was an elderly patient with a history of coronary artery disease, two other risk factors for asymptomatic gangrenous cholecystitis [21]. In the majority of reported cases of synchronous appendicitis and cholecystitis, right upper quadrant (RUQ) tenderness was present [2, 4–7, 10, 12–14].

### Imaging findings

This patient had both US and CT imaging on presentation. The final US report noted mild fatty infiltration of the liver, multiple gallbladder calculi and a 15 mm non-mobile calculus in the neck with asymmetric thickening of the wall, measuring up to 4.5 mm. No pericholecystic oedema was seen. However, the patient had a positive sonographic Murphy’s sign, and this finding in conjunction with the wall thickening was suggestive of cholecystitis.

CT of the gallbladder was initially reported as showing gallstones with a slightly thickened wall and minor stranding, suggestive of chronic cholecystitis. On subsequent review of the imaging by a second radiologist, this was felt to be more consistent with acute cholecystitis with some areas of the wall showing no enhancement, in keeping with necrosis.

US is generally thought to be the gold standard of gallbladder imaging due to the increased sensitivity for stones of certain composition on US compared with CT. However, more recent studies have suggested that while US may have higher specificity, CT may have higher sensitivity for acute cholecystitis [22]. In reality, a finding of cholecystitis in either modality would usually be sufficient justification for laparoscopy provided there were no contraindications. However, this emphasizes the fact that in these cases both clinical exam and imaging findings may be falsely reassuring. Ultimately, laparoscopy is the most reliable way to assess the gallbladder.

### True synchronicity

Final histology suggested that both pathologies arose independently and synchronously. Gangrenous cholecystitis was likely secondary to ischaemia from the impacted stone and appendiceal perforation secondary to the fragile diverticula wall at the tip which perforated in the absence of a fecalith. No reported cases of synchronous appendicitis and cholecystitis describe diverticular perforation as the underlying etiology of appendicitis. Of note, this patient was of south Asian heritage, and right sided diverticulitis would also be an important differential. This case illustrates the importance of a broad differential and comprehensive panel of investigations, particularly in patients with Type 2 Diabetes and atypical symptoms.
